# Perioperative management of the vulnerable and failing right ventricle

**DOI:** 10.1186/s13741-024-00397-5

**Published:** 2024-05-16

**Authors:** R. C. Arora, J. K. Brown, S. Chatterjee, T. J. Gan, G. Singh, M. Z. Tong, on behalf of the POQI IX Consortium

**Affiliations:** 1 Harrington Heart and Vascular Institute - University Hospitals, Cleveland, OH USA; 2 Department of Anesthesiology and Perioperative Medicine, University of Texas MD Anderson Cancer Center, Houston, TX USA; 3 Department of Surgery, Baylor College of Medicine, Houston, TX USA; 4 Division of Anesthesiology and Perioperative Medicine, Critical Care and Pain Medicine, University of Texas MD Anderson Cancer Center, Houston, TX USA; 5 Mazankowski Alberta Heart Institute, Edmonton, AB Canada; 6 Heart, Vascular and Thoracic Institute, Cleveland Clinic, Cleveland, OH USA; 7 Department of Surgery, Case Western Reserve University, Cleveland, OH USA; 8 Department of Cardiovascular Surgery, Texas Heart Institute, Houston, TX USA; 9 Departments of Critical Care Medicine and Surgery, University of Alberta, Edmonton, AB Canada

**Keywords:** Right ventricle, Heart failure, Right heart failure, Management, Scoring system

## Abstract

Under recognition combined with suboptimal management of right ventricular (RV) dysfunction and failure is associated with significant perioperative morbidity and mortality. The contemporary perioperative team must be prepared with an approach for early recognition and prompt treatment. In this review, a consensus-proposed scoring system is described to provide a pragmatic approach for expeditious decision-making for these complex patients with a vulnerable RV. Importantly, this proposed scoring system incorporates the context of the planned surgical intervention. Further, as the operating room (OR) represents a unique environment where patients are susceptible to numerous insults, a practical approach to anesthetic management and monitoring both in the OR and in the intensive care unit is detailed. Lastly, an escalating approach to the management of RV failure and options for mechanical circulatory support is provided.

## Introduction

Right ventricular (RV) function has garnered increased attention due to poor postoperative outcomes in patients with acute RV failure and chronic RV dysfunction with pulmonary hypertension (PH) (Ren et al. 2019). Both pressure and volume overload alter RV mechanics and function (Konstam et al. 2018). RV dysfunction (RVD) refers to impaired RV filling or ejection not associated with overt symptoms of heart failure (Harjola et al. 2016). RV failure (RVF) describes reduced forward flow through the pulmonary circulation resulting in a low cardiac output (CO) state, evidenced by hypotension, cool extremities, and systemic congestion (jugular venous distension, peripheral edema, oliguria, and congestive hepatopathy) (Konstam et al. 2018; Jabagi et al. 2022; Hrymak et al. 2017).

RVF can be further characterized as acute or chronic. Acute RVF is a “progressive, often rapid, syndrome characterized by systemic congestion secondary to impaired RV filling and/or reduced RV flow output” (Harjola et al. 2016). Chronic RVF generally results from increased RV afterload caused by PH, most frequently from left ventricular (LV) failure, although pulmonary arterial hypertension, lung disease, and chronic volume overload from right-sided lesions such as tricuspid regurgitation are also known etiologies.

### Why it matters

RVD and RVF are associated with significant morbidity and mortality following major surgery and severe respiratory illness (Hrymak et al. 2017; Haddad et al. 2009; Raina and Meeran 2018; Sato et al. 2021). Clinicians must integrate clinical examination with monitoring data for early recognition and diagnosis, identify precipitating etiologies, and rapidly initiate management to reverse or attenuate RVF. This review aims to increase awareness of methods to optimize RV function throughout the perioperative course, utilizing a proposed scoring system to aid risk assessment and resource allocation.

## Methods

The Perioperative Quality Initiative (POQI) is a non-profit organization that assembles international, multidisciplinary teams to develop consensus-based perioperative medicine recommendations (Workgroup et al. 2016). POQI methodology combines evidence appraisal and expert opinion, acknowledging available literature limitations and providing practical guidance. The ninth POQI meeting and Cardiac ERAS Society convened between December 1 and 2, 2022 (New Orleans, LA, USA) to address RV management for surgical patients.

Three separate working groups with expertise in physiology, assessment, and RV management were created. Groups were assembled beforehand, including multidisciplinary representation (surgery, anesthesiology, nursing, and critical care). International experts were recruited (the USA, Canada, and the UK). Led by a chair, the group members performed individual electronic literature searches, generating bibliographies. Questions regarding controversies and lack of consensus were identified by the group members, then compiled and finalized by the chair for the RV Management Group (RCA). In advance, the literature was assembled and reviewed across the working group. Literature sampling and selection were performed through a PubMed search using the terms “right ventricular dysfunction,” “right ventricular failure,” and “major surgery.” Case reports, editorials, commentaries, or non-English language articles were excluded. A shared knowledge process constructed recommendations, a modification of the previously described POQI consensus statements (Workgroup et al. 2016). During the conference, questions were refined, and recommendations were developed via four plenary sessions and three small group sessions. Voting was conducted openly during plenary sessions, which included the entirety of conference attendees reviewing each working group’s statements. While influence and bias cannot be completely excluded during voting, moderators actively encouraged dissenting opinion presentations. Diverging opinions were recognized and debate expanded group knowledge and awareness, informing the drafted consensus statement. These iterative sessions solidified consensus statements. During the final session, all members from all three working groups voted to agree or disagree with the statements and proposed paradigms.

### Proposed paradigm

Acute RV decompensation may be swift and dramatic. As such, a framework for determining potential patient needs should be based on the context of planned surgical intervention, monitoring considerations, potential treatments, facility resources, and personnel. The proposed scoring system provides a pragmatic approach for expeditious decision-making.

**Fig.1:**
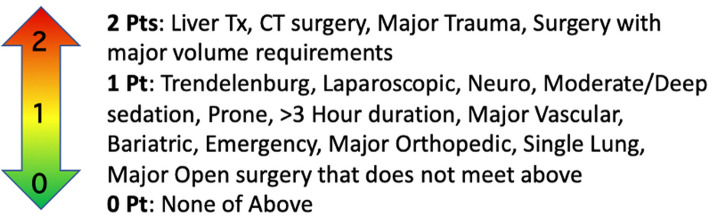
Following consideration of the stress of surgery, RV function should be ascertained. This is graded 1–5 from minimal risk (1 point) up to refractory shock (5 points) (Fig. 2). The sum of the stress of surgery (0–2) and the baseline level of RV function (1–5) generates the cumulative POQI 9 RV Risk Score, which has 5 levels. The higher the level, the greater the potential for escalating care requirements (see Fig. 2)

The anesthetic resources required to identify, monitor, and treat patients at risk for RV decompensation during surgery begin with recognizing the stress of surgery on the right ventricle, graded from 0 to 2. This risk may be due to physiologic stress, e.g., pneumoperitoneum, single-lung ventilation, potential for large volume transfusion, or the nature of the procedure itself. Moderate risk such as the need for Trendelenburg positioning or laparoscopic surgery is assigned 1 point. Surgeries at the highest risk of RV decompensation are assigned 2 points, including liver transplantation, cardiothoracic surgery, major trauma, and procedures necessitating large volume replenishment (Fig. 1).

### Anesthetic management

#### Level of care determination

**Fig. 2:**
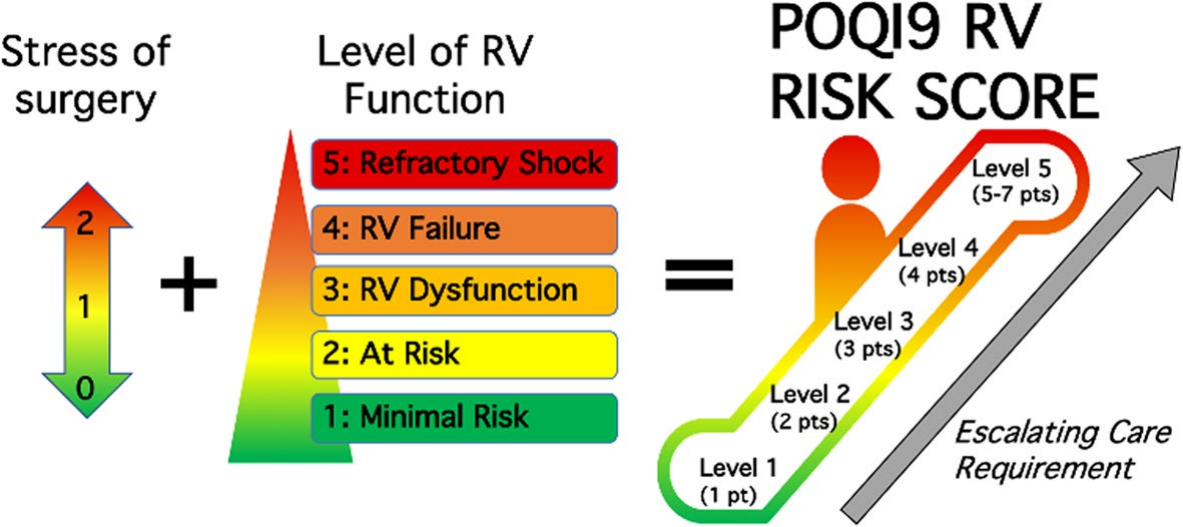
Once the POQI 9 RV Risk Score is determined, a guide for the level of anesthetic monitoring and potential treatment is proposed (see Table 1)

Anesthetic care must be tailored to RVD severity coupled with the impact of the surgical stress response. The “POQI 9 RV Risk Score” can guide monitoring techniques and management (Fig. 2 and Table 1). Healthcare providers with expertise in the assessment and management of RVD are essential to ensure optimal perioperative care.

**Table 1:** POQI 9 RV Risk Score and clinical activity

POQI 9 RV Risk Score	Notes and examples	Potential treatments	Monitoring tool considerations	Additional facility capabilities
**Level 1** (1 point)	-*No RVD risk (1 point) undergoing low-stress surgery (0 points)*	-Best anesthetic practice	-ASA standard monitoring	
**Level 2** (2 points)	-Overall, low-risk patients *-May have RVD risk factors (2 points) undergoing lower-risk surgery (0 points)* *-Patient with risk factors for RVD (1 point) undergoing moderate-risk surgery (1 point*)	-Preventative measures -Avoid hypoxia, hypotension, hypervolemia, hypercapnia, acidosis, and dysrhythmias -Lung protective ventilation	-Consider arterial line	
**Level 3** (3 points)	-Require increased monitoring capabilities and require planning for higher levels of treatments for potential RV decompensation *-Patient without risk factors (1 point) undergoing a liver transplant (2 points)* *-Known RVD risk factors (2 points) undergoing lobectomy (1 points)*	-Preventative measures described for level 2 patients -Diuretics -Low-dose inodilators	-Consider arterial line -Consider CVC	-Consider post-op care in a unit capable of higher monitoring
**Level 4** (4 points)	-Moderate to high risk of RV decompensation -Anticipate the need for RV support *-Known RVD (3 points) undergoing laparoscopic cholecystectomy (1 point)* *-Known risk factors (2 points) undergoing major cardiac surgery (2 points)*	-Inotropes -Vasopressor -Inhaled pulmonary vasodilators -Continuous renal replacement therapy (CRRT)	-Arterial line -CVC -Consider other advanced hemodynamic monitoring -Consider transesophageal echocardiography (TEE)	-ICU -Possible ECMO initiation capability
**Level 5** (5–7 points)	-Either in refractory shock or high risk of RV *-Left ventricular assist device (LVAD) patients with known RVF (4 points) needing hemicolectomy (1 point)* *-Acute PE with RV strain (4 points) undergoing pulmonary embolectomy (2 points)*	-ECMO -RVAD	-Arterial line -CVC -Consider other advanced hemodynamic monitoring (PAC) -Transesophageal echocardiography (TEE)	-Specialized advanced heart failure team and resources

The identification and optimization of modifiable RVD risk factors are crucial. Time permitting, medications, oxygen therapy, and continuous positive airway pressure (CPAP) devices may be used to improve underlying RV and LV function or pulmonary disease (Steppan and Heerdt 2021). RVF can be further mitigated through preoperative volume optimization and judicious surgical techniques to minimize bleeding and ensure myocardial protection during cardiac surgery (McGlothlin et al. 2022).

#### Anesthetic technique

The OR represents a unique environment where patients are susceptible to numerous insults including hypotension, volume shifts, and rhythm disturbances. Anxiety, pain, hypothermia, hypoxemia, hypercarbia, and acidosis contribute to hemodynamic derangements. While most patients tolerate these perturbations, the consequences in RVD patients can be profound.

There is no ideal anesthetic technique for patients with RVD; thus, the choice of monitored anesthesia care (MAC) and regional or general anesthesia (GA) should be patient- and case-specific (Wanner and Filipovic 2020). Regardless, the physiologic goals are the same: maintain sinus rhythm and decrease RV afterload while optimizing RV preload, contractility, and perfusion (McGlothlin et al. 2012).

#### Regional anesthesia

Regional anesthesia is generally a safe option, whereby spontaneous ventilation avoids pulmonary pressure elevation from mechanical ventilation (Sarkar and Desai 2018). Anticoagulation status, the ability of the patient to lie flat, and the need for additional sedation are essential considerations (Wood et al. 2021). Epidural or combined spinal-epidural anesthesia with incremental administration of local anesthetic is preferred over spinal anesthesia to minimize bradycardia and systemic vascular resistance (SVR) reduction and avoid ensuing decreased preload, CO, perfusion pressure, and RV contractility (Wink et al. 2019).

#### Monitored anesthesia care (MAC)

MAC is the delivery of sedative and/or analgesic medications, titrated to a desired effect ranging from minimal to moderate and to deep sedation (Das and Ghosh 2015). MAC is thought to result in less physiologic disturbance than general anesthesia and is preferred for minor surgical procedures; however, it is not without risk (Krakowski and Arora 2021). Avoidance of oversedation is critical. Repeated or escalating doses of medication can lead to hypoventilation, thereby precipitating hypoxemia and respiratory acidosis, all of which increase pulmonary vascular resistance (PVR) (Smeltz and Kumar 2021).

#### General anesthesia

Induction is the most hemodynamically vulnerable phase of anesthetic care (Zucker et al. 2022). Increased sympathetic tone and apneic periods during direct laryngoscopy, combined with hypotension and myocardial depressant effects of many induction agents, make preparation and a well-thought-out plan crucial. Propofol, for example, decreases SVR, mean arterial pressure (MAP), and venous return, contributing to significant hypotension (Reich et al. 2005). The use of ketamine in PH and RVD has been debated. The combined analgesic and hypnotic properties in the absence of myocardial depression and hypotension may outweigh the small potential increase in PVR (Maxwell and Jackson 2012). Etomidate has minimal hemodynamic impact and may decrease PVR, making it ideal in this patient population (McGlothlin et al. 2022). Regardless of the induction agent, slow, incremental medication titration is essential. To date, no evidence supports inhalation versus total intravenous anesthetics (TIVA) for the maintenance of anesthesia (Wood et al. 2021). The use of nitrous oxide is discouraged, however, as it can cause a mild increase in PVR and worsen hypoxemia (Schulte-Sasse et al. 1982). This is in direct contradistinction to nitric oxide. Once general anesthesia is established, mechanical ventilation strategy, medical management, and the selection of hemodynamic monitoring tools are critical to mitigate development of acute RVF.

### Intraoperative management

#### Ventricular preload

Preload must be carefully balanced to ensure CO and minimize RV overdistension. A previous misconception was that RV dysfunction and failure should be treated with volume supplementation, presuming the RV is a passive conduit. Despite the ability to tolerate a higher preload and adequate right-sided filling pressure is necessary to initially preserve CO, patients with RVD can develop RV volume overload resulting in RVF (Varma et al. 2022). RV distension shifts the interventricular septum towards the LV, compromising LV stroke volume. Due to ventricular interdependence, RV overdistension leads to worsening tricuspid regurgitation, increasing wall tension, decreasing contractility shifting the interventricular septum leftward, compromising LV filling, and worsening CO and organ dysfunction (Arrigo et al. 2019; Mahmood and Pinsky 2018; Green and Givertz 2012). Invasive monitoring such as TEE, continuous pulse pressure evaluation, and/or pulmonary artery catheters should be considered to help guide fluid management.

#### Ventricular afterload/mechanical ventilation

RV systolic function is highly sensitive to changes in afterload as determined by PVR; minor increases in PVR from hypoxemia, hypercarbia, and acidosis as seen with anxiety, pain, and hypothermia cause large decreases in stroke volume (Konstam et al. 2018). Ventilator management strategies strive to avoid increases in PVR that can be seen both at high lung volumes, when intra-alveolar vessels become compressed and at low lung volumes, when hypercarbia and hypoxic pulmonary vasoconstriction occur (McGlothlin et al. 2022; Strumpher and Jacobsohn 2011). Optimum PEEP improves oxygenation by recruiting alveoli yet avoiding hyperinflation that results in shunting and worsening of ventilation-perfusion mismatch (see Table 2) (Haddad et al. 2009).

**Table 2:** General clinical management parameters for perioperative RV management

Parameter and goal	Rationale
PaCO_2_ = 30– 35 mmHg	Avoid hypercarbia-induced pulmonary vasoconstriction
pH ≥ 7.4	Avoid acidosis-induced pulmonary vasoconstriction
Plateau pressures < 27–30 cmH_2_O with low tidal volumes (6–8 mL/kg predicted body weight) (McGlothlin et al. 2022; Arrigo et al. 2019; Strumpher and Jacobsohn 2011)	Minimize intrathoracic pressure-induced PVR elevation
SaO_2_ > 92%	Adequate oxygenation to prevent hypoxia-induced pulmonary vasoconstriction
Optimize PEEP	Improve alveolar recruitment but avoid hyperinflation (aim 5–10 cmH_2_O)

#### Monitoring

In addition to the American Society of Anesthesiologists (ASA) standard monitoring, advanced monitoring devices should be considered and capabilities escalated based on the level of RVD and surgical stress, as guided by the POQI 9 RV Risk Score (Table 1).

### Arterial catheter

These devices are warranted in most RVD patients in order to provide continuous blood pressure monitoring and arterial blood gas measurement. For stable RVD patients undergoing minor procedures with local or light sedation, arterial catheters may not be necessary. However, in high-risk patients or complex surgeries, catheters should be considered prior to anesthesia induction.

### Central venous catheter (CVC)

The CVC offers dependable intravenous access when vasopressors, inotropes, and pulmonary vasodilators are needed. Though not an accurate measure of RV preload, changing trends or sudden changes in CVP should trigger an investigation.

### Pulmonary artery catheter (PAC)

PACs may be useful adjuncts to guide fluid management and titrate vasoactive medications in selective high-risk patients when significant hemodynamic disturbances are expected; however, their utility has been debated (Wanner and Filipovic 2020). Monitoring trends in CVP, pulmonary artery pressure (PAP), CO, and mixed venous oxygen saturation (SvO_2_) can provide useful feedback, but interpretation requires a thorough understanding (Tilea et al. 2021; Marik 2013).

### Transesophageal echocardiography (TEE)

Expeditious evaluation is possible with transthoracic ultrasound (in MAC or regional cases) or TEE during general anesthesia but requires an experienced operator. TEE provides valuable information by enabling serial assessment of RV performance, fluid status, and assessment of other etiologies (Haddad et al. 2009; Silverton et al. 2022).

### RV decompensation

Timely recognition of RV decompensation depends on the expeditious interpretation of available laboratory, ventilation, and hemodynamic data. For a detailed evaluation of RV decompensation, please refer to the POQI 9 RV Assessment Group Recommendations (*reference to be inserted pending acceptance*). Management typically begins by analyzing volume, pressure, and contractility and addressing reversible causes. Elevations in right atrial pressure (RAP) above 5–10 mmHg (McGlothlin et al. 2022) or 8–12 mmHg may necessitate RV afterload reduction (nitroglycerin, inhaled pulmonary vasodilators) or judicious use of intravenous diuretics. If unresponsive and hemodynamics remain poor, hemofiltration may be necessary. At the low range of RAP, fluid boluses should be delivered in small aliquots (100–250 mL) to avoid rapid RV overdistension (Harjola et al. 2016; Green and Givertz 2012; Marik 2015). Systemic hypotension worsens RV coronary perfusion and contractility (Wolferen et al. 2008). The recommended MAP target is greater than 65 mmHg (Ruetzler et al. 2021). Vasopressin and norepinephrine are preferred over phenylephrine (Strumpher and Jacobsohn 2011). Combinations of inotropes, inodilators, and intravenous vasodilators may be required. There is insufficient evidence to demonstrate superiority of one medication over the other, but first-line vasoactive drugs to stabilize hemodynamics include dobutamine, milrinone, epinephrine, and levosimendan (though not currently available in North America) (Price et al. 2021). Both milrinone and levosimendan can produce systemic vasodilation, and their use will likely require the addition of vasopressors to support systemic blood pressure (Wanner and Filipovic 2020; Navaratnam and DiNardo 2020; Forrest 2009). Inodilators have the additional benefit of reducing PVR.

#### Arrhythmias

Attempt electrical or pharmacological cardioversion with amiodarone. Atrial pacing can be used for bradycardia (see also the “Optimizing rate and rhythm” section) (Kapur et al. 2017).

#### Persistent RVF

Selective pulmonary vasodilators cause less systemic hypotension than systemic pulmonary vasodilators and should be used while optimizing hemodynamics. Options depend upon institutional availability and include inhaled nitric oxide (5–40 ppm continuously), inhaled milrinone (2–5 mg) for 10–15 min, or inhaled prostacyclin analogs such as iloprost (5–10 mg, nebulized), prostacyclin (25–50 mcg nebulized), and inhaled epoprostenol (10–50 ng/kg/min continuous) (Strumpher and Jacobsohn 2011). Prompt and open communication with all stakeholders, including the surgical team to discuss the need to abort the procedure if appropriate, the ICU team for postoperative management, and possible consultation with the MCS team for backup mechanical support should be discussed.

### Postoperative/ICU management of RVF

#### Postoperative disposition

Postoperatively, patients failing to fulfill the post-anesthetic care unit (PACU) discharge criteria necessitate consideration for ICU care for ongoing mechanical ventilation, vasoactive support, and/or closer hemodynamic monitoring. Transfer is appropriate when ongoing care needs exceed the institutional level of care capacity. The POQI 9 RV Risk Score aids in planning for such contingencies.

#### General overview of RVF management

**Fig. 3:**
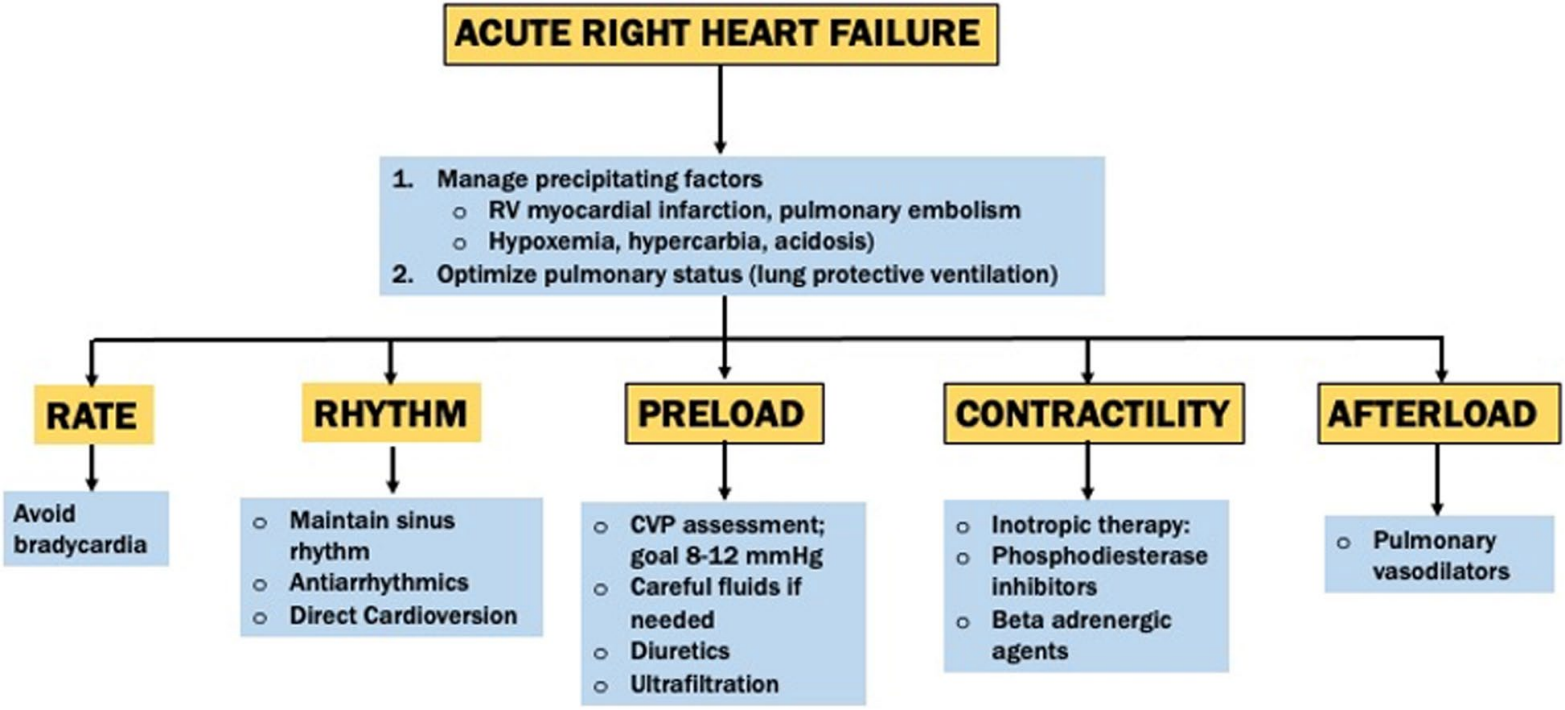
An overview of the management algorithm for the acutely failing RV

Similar to pre- and intraoperative planning, interdisciplinary cooperation is essential to successful postoperative management and to facilitate escalation to higher-intensity therapies, when indicated. Several objective criteria can be utilized to guide interventions, such as cardiac filling pressures [CVP and pulmonary capillary wedge pressure (PCWP)], pulmonary artery pulsatility index (PAPI), and transpulmonary gradient (TPG) (Kapur et al. 2017). Increased monitoring includes PAC, which may be necessary to appropriately implement therapies. Acute RVF assessment begins by identifying potentially treatable conditions. This may include coronary revascularization for acute coronary syndrome or thrombolytic therapy for acute pulmonary embolism (Kapur et al. 2017). Early RVF recognition as a result of systemic hypoperfusion, low-pulse pressure, elevated CVP, and end-organ malperfusion is essential. Evaluating the underlying etiology and specific treatment implementation may be indicated. Basic principles include maintaining adequate CO and treatment of LVF. An overview of management is listed in Fig. 3.

### Conceptually: rate, rhythm, preload, contractility, and afterload

#### Optimizing rate and rhythm

Sinus rhythm and atrioventricular synchrony maintenance are required for optimal RV performance (Haddad et al. 2009). Atrial fibrillation is poorly tolerated due to loss of the atrial contribution to cardiac output. Early amiodarone administration, electrolyte correction, and electrical cardioversion should be considered (Hrymak et al. 2017). Generally, a higher HR (80–100 bpm) reduces RV filling, distension, pressure, and tricuspid regurgitation. In cardiac surgical patients, atrial pacing may be used.

#### Maintenance of systemic and RV coronary perfusion

Maintenance of systemic blood pressure with a minimal MAP target of 65–70 mmHg should be achieved. As RV pressures become elevated, the normal RV perfusion occurring in systole and diastole results in more occurring in diastole. Hypotension is treated with norepinephrine or vasopressin as first-line agents. Further, abdominal perfusion or organ perfusion pressure measured as MAP–CVP should be targeted to remain above 60 mmHg for adequate perfusion of intra-abdominal organs. As a result, patients with higher CVP from RVF may need a higher targeted MAP to satisfy this goal (Arrigo et al. 2019).

### Medical management

#### Optimization of preload

As with intraoperative management, avoidance of RV overdistension is a key element of postoperative RVD/F management. Typically, a CVP goal of 8–12 mmHg is targeted. In addition, mixed venous oxygen saturation (SvO_2_) that is preserved (> 65%) and normal lactate provide assurance that cardiac output is adequate (Harjola et al. 2016).

The first line for volume overload is diuretics when presenting with signs of venous congestion. Loop diuretics and sodium excretion can be used to achieve optimal preload. Since intravenous loop diuretics are most effective within the first few hours, the effect is completed in 6–8 h, three to four daily doses, or a continuous infusion may be necessary for optimal diuresis (Arrigo et al. 2019).

In the event that diuresis is inadequate to achieve fluid status goals, or there is evidence of azotemia or acute kidney injury with indications for renal replacement therapy, then ultrafiltration (UF) should be initiated to achieve volume removal. In patients with acute decompensated heart failure, UF is beneficial compared to loop diuretics with regard to decongestion and clinical improvement but has not yet been demonstrated to change rates of rehospitalization or improve survival (Kabach et al. 2017).

### Contractility/inotropes

**Fig. 4:**
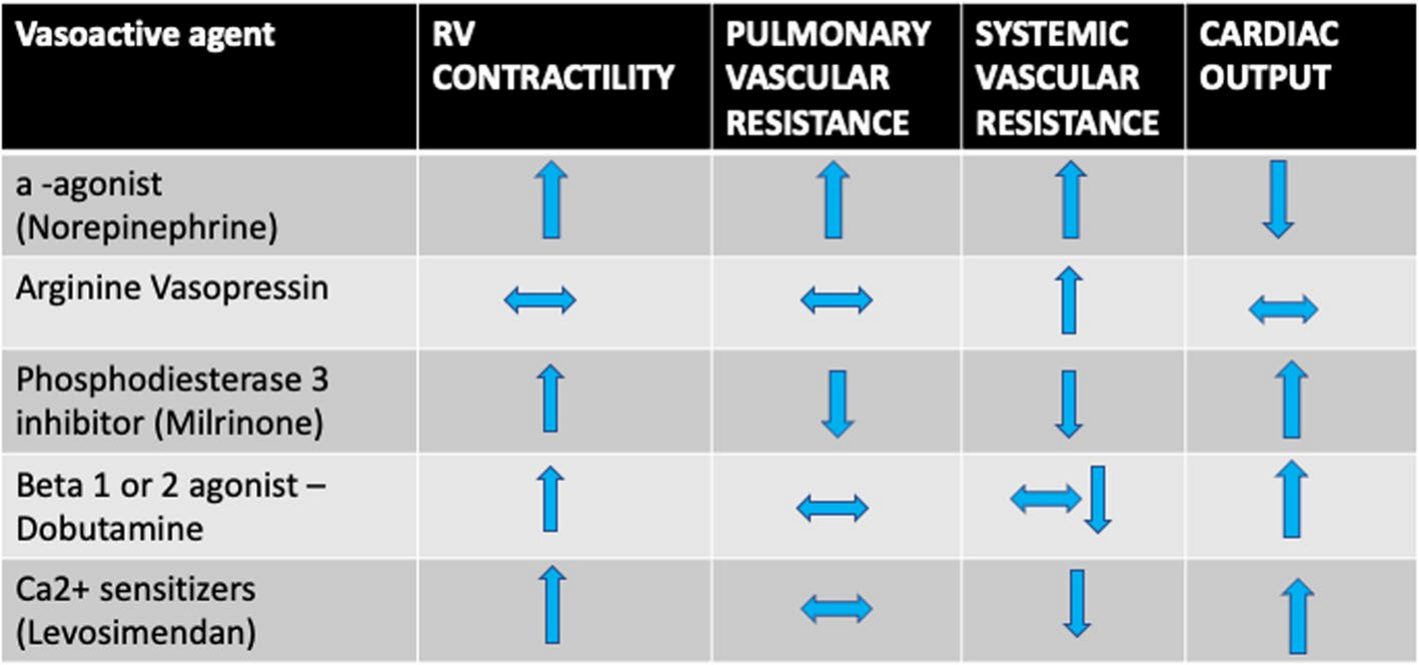
Summary of vasoactive agents

Preferred agents include milrinone, a phosphodiesterase inhibitor and a inodilator; dobutamine, a B1 agonist with minimal alpha and beta 2 agonist activity; and levosimendan, a calcium-sensitizing inotrope, which is currently unavailable in North America (McGlothlin et al. 2022). In hypotensive patients, epinephrine is a consideration. Inotropes are an appropriate intervention in RVD causing decreased LV stroke volume or cardiogenic shock. The use of mixed venous oxygen saturation can help determine the adequacy of perfusion (King et al. 2014). A summary of the most common agents and their hemodynamic effects is outlined in Fig. 4.

### Afterload reduction/pulmonary vasodilators

Pulmonary vasodilators or a combination of vasodilators for elevated PVR may be necessary. Inhaled pulmonary vasodilators, discussed above, include a prostacyclin (eproprostenol), two prostacyclin derivatives (treprostinil and iloprost), or nitric oxide, a soluble guanylase cyclase stimulator (Liu et al. 2021; Hoeper and Granton 2011).

### Mechanical ventilation

Hypoxemia, hypercapnia, and acidosis elevate PVR, with deleterious consequences. Cardiopulmonary interactions can adversely affect hemodynamics, with rapid deterioration ensuing. Lung protective ventilation, as reviewed above, can optimize hemodynamics by minimizing cardiopulmonary interactions (McGlothlin et al. 2022; Haddad et al. 2009). Targeting tidal volumes 4–6 mL/kg of predicted body weight (PBW) will prevent large tidal volumes and elevated intrathoracic pressure that can exacerbate PVR (Hoeper and Granton 2011). It is critical, however, that hypercapnia and ensuing respiratory acidosis be avoided. Accordingly, starting with 6–8 mL/kg PBW and gradually reducing tidal volumes while increasing respiratory rate, to maintain or increase minute ventilation, may be the safest strategy (King et al. 2014).

### Mechanical circulatory support (MCS)

When RV decompensation is refractory to medical management, consideration of escalation to MCS becomes imperative. This decision necessitates balancing procedural risks (bleeding, thrombosis) against the risk of continued RVF despite maximal medical management (irreversible end-organ malperfusion, death). Timing is critical, and generally, earlier MCS escalation for RVF offers superior survival (Kapur et al. 2017).

Intra-aortic balloon pump is the most widely employed MCS device. Although designed primarily for LV support, RV function can improve through both increased coronary blood flow and LV unloading. The overall CO increase provided by IABP is modest but may be sufficient in patients only requiring minimal support.

Extracorporeal membrane oxygenation (ECMO) is increasingly available and the easiest to establish among the various device options. It is well-suited for patients in extremis or at centers with limited MCS options. Once stabilized, the RV is unloaded and adequate systemic flow is provided. While providing circulatory support, ECMO does not unload the LV and increases LV afterload. Accordingly, in severe LV failure, ECMO can paradoxically contribute to RV dilatation by increasing left ventricular end-diastolic pressure (LVEDP) and pulmonary pressures. Assessing cardiac loading conditions with echocardiography or PAC is critical. LV decompression is essential when cardiac distension develops (Kapur et al. 2017).

Percutaneous right ventricular assist device (RVAD) options include dual lumen single-cannula RV support either with Protek Duo (LivaNova, London, UK) or Spectrum (Spectrum Medica, Gloucester, UK), or a micro axial pump, such as the Impella RP Flex (Abiomed. Danvers, MA). These devices can be inserted in the upper extremity veins, permitting patient ambulation. However, imaging guidance for insertion is mandatory and best performed in the cardiac catheterization lab or the operating room by trained operators. These devices are best suited for patients with declining hemodynamics but not yet manifesting more profound metabolic derangement secondary to prolonged shock states (Harjola et al. 2016).

Finally, for postcardiotomy shock following cardiac surgery, RVAD can be established by sewing a graft onto the pulmonary artery attached to the return limb of the ECMO circuit and inserting a venous cannula for the drainage limb of the ECMO circuit (Itagaki et al. 2012; Estrada et al. 2016; Saxena and Marasco 2015).

Device selection ultimately depends on center expertise and available devices, imaging service access, whether the chest is open or closed and, most importantly, the severity of cardiogenic shock. Institutions with limited capabilities for device deployment or ongoing care capabilities should partner with advanced heart failure centers (Itagaki et al. 2012).

### De-escalation

Exit strategies should be incorporated into any decision for MCS rescue. Once cannulated for ECMO, Biventricular VAD (BiVAD), or RVAD, the etiology of RVF determines the exit strategy pathway. Patients with acute RVF due to insults such as the stress of surgery will often recover following a period of support. Conversely, for patients with acute or chronic RVF, with pre-existing severe pulmonary vascular disease or severe LV failure, treatment must focus on PH or LV dysfunction to maximize the likelihood of RV recovery (Kapur et al. 2017).

Weaning strategies are device-specific and beyond the scope of this paper. In general, for ECMO patients, we advocate a transition to a tailored univentricular support system, permitting the coupling of LV and RV support (Randhawa et al. 2021). RVAD patients can generally be weaned, preferably guided by either use of a PAC or serial echocardiography (Harjola et al. 2016).

Lastly, some patients may not recover RV function and may not be candidates for therapeutic escalation. Palliative care consultation is recommended for appropriate patients and family support (Hoeper and Granton 2011).

### Controversies and future research needed

While our physiologic understanding has improved with diagnostic assessment and clinical management of RVD, numerous areas merit investigation. These include more accurate and minimally invasive hemodynamic monitors. Improved ventilator management may incorporate alternative ventilation strategies that improve RV hemodynamics. Perhaps earlier inhalation of inhaled pulmonary vasodilators using non-invasive ventilation may be appropriate in some patients. In specific settings, for example, massive pulmonary embolism, earlier initiation of ECMO may allow for RV rest and recovery without additional therapies beyond systemic anticoagulation. Lastly, improved pharmacotherapies and vasoactive medications require evaluation.

## Summary

Caring for the patient with right ventricle (RV) dysfunction remains a management challenge and inadequate support results in poor postoperative outcomes. Perioperative care must be tailored to the degree of RV dysfunction coupled with the impact of the surgical stress response. The provided POQI 9 RV Risk Score provides a framework for the perioperative team to consider in patient selection, timing of surgery, appropriate monitoring, and management of these complex patients. In regard to the monitoring and management of the patient RV dysfunction or failure, it is recommended that the perioperative team consider if their institution has the appropriate capacity and expertise to provide comprehensive care for these potentially challenging patients. Optimal therapies remain controversial, and it is acknowledged that additional research, pharmacotherapies, and mechanical circulatory support devices are needed.

## Data Availability

No datasets were generated or analysed during the current study.

## Abbreviations

BiVAD: Biventricular assist device
CO: Cardiac output
CVC: Central venous catheter
CPAP: Continuous positive airway pressure
ECLS: Extracorporeal life support
ECMO: Extracorporeal membrane oxygenation
GA: General anesthesia
HR: Heart rate
LV: Left ventricle
MAC: Monitored anesthesia care
MCS: Mechanical circulatory support
OR: Operating room
PAC: Pulmonary artery catheter
PAPI: Pulmonary artery pulse index
PEEP: Positive end-expiratory pressure
PVR: Pulmonary vascular resistance
RA: Right atrium
RAAS: Renin–angiotensin–aldosterone system inhibitors
RAP: Right atrial pressure
RV: Right ventricle
RVAD: Right ventricular assist device
RVD: Right ventricular dysfunction
RVF: Right ventricular failure
SvO2: Mixed venous oxygen saturation
SVR: Systemic vascular resistance
TIVA: Total intravenous anesthetics
